# Quantifying endothelial damage by digital droplet polymerase chain reaction (PCR) of endothelial cell-free DNA in COVID-19 patients

**DOI:** 10.1016/j.rpth.2025.103320

**Published:** 2026-01-13

**Authors:** Tiphaine Ruggeri, Gertrud Wiedemann, Noëlia Schärz, Barbara Hügli, Andreas Limacher, Cédric Hirzel, Naomi Porret, Sacha Zeerleder

**Affiliations:** 1 Department of Hematology and Central Hematology Laboratory, Inselspital, Bern University Hospital, Bern, Switzerland; 2 Department for BioMedical Research, University of Bern, Bern, Switzerland; 3 Center for Laboratory Medicine, University Institute of Clinical Chemistry, Inselspital, Bern University Hospital, Bern, Switzerland; Department of Clinical Research, University of Bern, Bern, Switzerland; 4 Swiss Paraplegic Research, Nottwil, Switzerland; 5 Department of Infectious Diseases, Inselspital, Bern University Hospital, University of Bern, Bern, Switzerland; 6 Department of Hematology, Kantonsspital Luzern, Lucerne, Switzerland; University of Bern, Bern, Switzerland; 7 Faculty of Health Sciences and Medicine, University of Lucerne, Lucerne, Switzerland

**Keywords:** cell-free nucleic acids, COVID-19, digital PCR, endothelial cells, methylation, microcirculation, nitric oxide synthase type III, thrombosis

## Abstract

**Background:**

COVID-19, caused by SARS-CoV-2, triggers severe systemic inflammation and multiple organ dysfunction. Microvascular complications, potentially arising from endothelial cell infection and/or immunothrombosis, play a central role in the disease's pathophysiology. Upon cell activation and/or cell death, cells release cell-free DNA (cfDNA) into the circulation, and cfDNA derived specifically from endothelial cells may serve as a marker of microvascular damage severity.

**Objectives:**

In this study, we aimed to develop an assay to specifically measure endothelial cell-derived DNA as a marker of microvascular damage in COVID-19 patients.

**Methods:**

In this study, we developed a methylation-specific digital droplet polymerase chain reaction assay targeting the promoter of the *NOS3* gene to quantify circulating endothelial cell-derived cfDNA in COVID-19 patients followed longitudinally at inclusion, day 11, and day 28.

**Results:**

Total cfDNA and endothelial-specific cfDNA levels significantly increased with COVID-19 disease severity, with the highest levels in patients with severe COVID-19. Notably, patients with mild COVID-19 showed endothelial cfDNA levels comparable to those of healthy controls, and levels remained stable from inclusion through day 28. In contrast, patients with moderate disease severity showed significantly elevated endothelial cfDNA levels compared with controls, which declined over time. Patients with severe COVID-19 displayed persistently high endothelial cfDNA levels throughout the observation period.

**Conclusion:**

Using a digital droplet polymerase chain reaction assay specific for cfDNA from endothelial cells, we demonstrated endothelial cell damage in patients with COVID-19 that correlated with disease severity.

## Essentials

• COVID-19 may cause microvascular injury by damaging endothelial cells.
• We monitored endothelial cell-free DNA in COVID-19 patients across 3 time points.
• Endothelial cell-free DNA rose with increasing disease severity and remained high in severe cases.
• The findings show that endothelial injury closely reflects COVID-19 severity and disease progression.

## 1 Introduction

Infection with severe SARS-CoV-2 causes COVID-19, which is characterized by systemic inflammation that leads to multiple organ dysfunction syndrome [1]. Prior to the availability of vaccines, specific antiviral drugs, and therapeutic neutralizing monoclonal antibodies for SARS-CoV-2, 14.5% of COVID-19 patients experienced severe systemic inflammation with subsequent organ failure despite viral clearance [1]. Based on postmortem analysis, microvascular complications are a main driver for the pathophysiology of multiple organ dysfunction syndrome in COVID-19. SARS-CoV-2 triggers endothelialitis and microthrombus formation, intensifying the inflammatory response [2,3].

Using electron microscopy, SARS-CoV-2 was detected in endothelial cells from different organs, including transplanted kidneys and skin lesions [3,4]. Studies of both animal models and humans have shown that SARS-CoV-2 can directly infect mature endothelial cells [5]. However, the notion that direct infection of endothelial cells is the predominant cause of microvascular complications has been challenged [6]. Histological data and biomarker-based evidence suggest that neutrophil activation, particularly in the form of neutrophil extracellular traps, occurs in patients with severe COVID-19 and contributes to microvascular complications [7]. However, the extent to which direct endothelial cell invasion by SARS-CoV-2 or neutrophil extracellular trap formation, potentially leading to immunothrombosis, independently or in combination, contributes to microvascular complications and subsequent organ dysfunction remains to be determined.

Upon cell activation and/or cell death, cells release cell-free DNA (cfDNA) into the circulation. The release of cfDNA is an active, highly regulated process [8,9]. Circulating cfDNA is a reliable marker for assessing severity and predicting fatal outcomes in conditions characterized by systemic inflammation, such as sepsis [[10], [11], [12]]. Total cfDNA levels are increased and correlate with disease severity in COVID-19 [[13], [14], [15]].

In this study, we developed and employed a methylation-specific digital droplet polymerase chain reaction (ddPCR) assay to quantify circulating cfDNA of endothelial cell origin in longitudinally followed COVID-19 patients. Using this approach, we aimed to determine whether the severity of COVID-19 correlates with endothelial cell damage.

## 2 Methods

### 2.1 Study design and participants

We enrolled 35 patients with polymerase chain reaction (PCR)-confirmed SARS-CoV-2 infection during the first 2 waves of COVID-19 from March 5 to December 15, 2020, at the University Hospital of Bern, Switzerland. SARS-CoV-2 vaccines were not yet available at that time. Blood samples were collected at enrollment (baseline), on day 11 ± 3 (D11), and on day 28 ± 7 (D28). COVID-19 disease severity was categorized according to the COVID-19 World Health Organization (WHO) Ordinal Scale for Clinical Improvement [16]. Patients with a score of 1 to 2 (outpatients) were classified as having mild disease, whereas patients with a score of 3 to 4 (admitted patients with or without oxygen by mask of nasal prongs) or 5 to 8 (noninvasive ventilation, high-flow oxygen, mechanical ventilation, extracorporeal membrane oxygenation, or death) were classified as having moderate or severe disease, respectively [16]. The present study is part of a larger COVID-19 project (NCT04510012), and data from some patients were previously analyzed for immune function and neuroaxonal damage [[17], [18], [19]]. This study was approved by the Ethics Committee of the Canton of Bern, Bern, Switzerland (number 2020-00877), and registered at ClinicalTrials.gov (NCT04510012). Patients were included after providing written informed consent. In cases of capacity limitations and/or inability to provide consent, enrollment followed the procedures for research projects in emergency situations according to Swiss law.

### 2.2 Sample collection

Blood from COVID-19 patients was drawn into S-monovette tubes containing 3.2% citrate (3 mL tubes, Sarstedt). Blood from healthy volunteers (*n* = 26 for measurement of cfDNA; *n* = 24 for measurement of nucleosomes) was obtained from the interregional blood donation center (Swiss Red Cross [SRK] AG in Bern, project number P357) using Vacuette tubes containing 3.2% citrate (3.5 mL tubes, Greiner Bio-One). All samples were centrifuged twice at 2500 × *g* for 15 minutes at room temperature. Plasma was then stored at −70 °C.

### 2.3 DNA extraction from cells

Genomic DNA (genDNA) from human dermal microvascular endothelial cells-1 (HMEC-1), human umbilical vein endothelial cells (HUVECs), and neutrophils (5 × 10^6^ cells) was extracted using the DNeasy Blood & Tissue Kit (Qiagen). For details on the HMEC-1 and HUVEC cultures and neutrophil isolation, see the Supplementary Methods. For the genDNA control, the buffy coats of 4 healthy donors from the interregional blood donation center (SRK AG in Bern, project number P014) were pooled. GenDNA was extracted using the QIAamp DNA Blood Mini QIAcube Kit (Qiagen) according to the manufacturer’s protocol.

### 2.4 cfDNA extraction from plasma

Extraction of cfDNA was performed using the QIAamp Circulating Nucleic Acid Kit (Qiagen). A sample volume of 500 μL citrated plasma was used and topped up to 1 mL with phosphate-buffered saline (pH 7.3, without calcium or magnesium) prior to extraction. cfDNA purification was performed according to the manufacturer's protocol, with a final elution volume of 25 μL AVE Buffer (Qiagen), and the eluate was stored at −20 °C.

As a negative control, cfDNA was extracted from 5 mL OctaplasLG (Octapharma AG), a pool of plasma from healthy donors with blood group B.

### 2.5 ddPCR

Details of DNA concentration measurement, DNA fragment analysis, and bisulfite conversion performed before ddPCR are provided in the Supplementary Methods. The workflow is summarized in Supplementary Figure S1. ddPCR targeting methylated or unmethylated sequences in the promoter region of the *NOS3* gene was performed using the QX200 Droplet Digital PCR System (Bio-Rad), with an AutoDG Automated Droplet Generator (Bio-Rad). Before ddPCR was performed, genDNA extracted from cells was digested with the restriction enzyme MseI (New England Biolabs) for 15 minutes at room temperature, a step not necessary for already small fragment-sized cfDNA samples. Amplification reactions for ddPCR were prepared according to the manufacturer’s protocol, adding 5 μL of bisulfite-converted DNA to 20 μL of the assay mix. The input template amount per ddPCR well was either half the cfDNA obtained from each sample after bisulfite conversion or 25 ng of genDNA after restriction digestion of cells, with all samples measured in duplicate. For droplet generation with the AutoDG Automated Droplet Generator, 20 μL of the reaction mixture was used per well. Amplification was performed using a C1000 Touch Thermal Cycler (Bio-Rad) with the following thermal conditions: 95 °C for 10 minutes, followed by 40 cycles of 94 °C for 30 seconds, a combined step for annealing and amplification at 58 °C for 1 minute, and a final deactivation step at 98 °C for 10 minutes. All steps were performed at a ramp rate of 2 °C/s. For readout, the QX200 Droplet Reader with the 2-color detection system set to FAM (channel 1) and HEX (channel 2) was used. Sequences and concentrations of primers and probes, as well as the ddPCR thermocycling conditions, are summarized in Supplementary Tables S1 and S2. The results were analyzed using QuantaSoft software (version 1.7.4.0917, Bio-Rad). For details, see the Supplementary Methods.

### 2.6 Nucleosome measurement

Nucleosome concentrations were quantified in EDTA plasma using an enzyme-linked immunosorbent assay method, as previously detailed in the literature [20]. In summary, enzyme-linked immunosorbent assay plates were initially coated with a monoclonal anti-histone H3 antibody (CLB/ANA-60). Plasma samples were then added and incubated for 1 hour at room temperature. Following a washing step, biotin-labeled F(ab’)2 fragments of a monoclonal anti-nucleosome antibody (CLB/ANA-58) were added and incubated for an additional hour at room temperature. The binding of the biotin-labeled antibodies was subsequently detected using streptavidin-horseradish peroxidase in conjunction with tetramethylbenzidine as the substrate. The enzymatic reaction was stopped with 2 M H_2_SO_4_, and the absorbance was measured at 450 nm.

### 2.7 Statistical analysis

Details of quantitative analysis are provided in the Supplementary Methods. The results are reported as median (IQR). In brief, for nonpaired analyses, we used the Mann–Whitney U-test or the Kruskal–Wallis test, followed by Dunn’s multiple comparison test. For paired analyses, we used the Wilcoxon signed-rank test. Correlations were assessed using Spearman’s rank correlation. Correction for multiple testing was performed using the Bonferroni method.

To evaluate differences in COVID-19 disease severity and cfDNA concentration, the different COVID-19 severity categories were compared using a nonparametric trend test according to Cuzick and a Wald test from a regression model with cluster-robust SEs [21]. The increase in the number of positive droplets across severity groups was calculated from a multivariate negative binomial model with 2 dependent variables and cluster-robust SEs to account for multiple samples per participant. The WHO Ordinal Scale for Clinical Improvement was used as an independent variable, and the number of droplets positive for endothelial cell-specific cfDNA and cfDNA from nonendothelial cell sources were used as dependent variables. Statistical significance was defined as *P* value < .05. Analyses were performed in Stata version 17 (Stata Corporation) and GraphPad Prism version 9.3.

## 3 Results

### 3.1 Development and optimization of a methylation-specific ddPCR assay for the detection of cfDNA originating from endothelial cells

**Figure 1:**
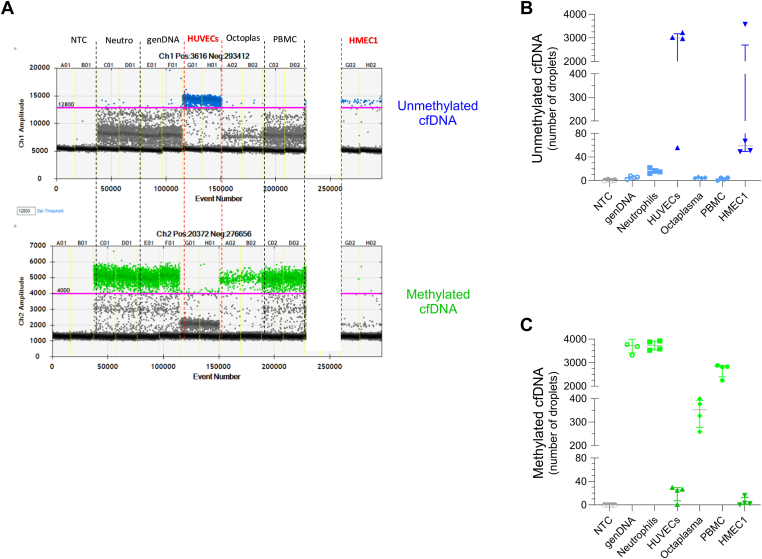
One-dimensional plots for methylation-specific digital droplet polymerase chain reaction (ddPCR) assay targeting NOS3 sequences in endothelial and blood cells. (A) One-dimensional plot view of QuantaSoft software showing the droplet signal of 7 controls and cell lines (no template control [NTC], neutrophils [Neutro], genomic DNA [genDNA], human umbilical vein endothelial cells [HUVECs], commercial pooled plasma [OctaplasLG; Octaplas], peripheral blood mononuclear cell [PBMC], and human dermal microvascular endothelial cells-1 [HMEC1]), which were used in all ddPCR experiments to assess the COVID-19 patients and healthy donor samples. HUVECs and HMEC1 are labeled in red, corresponding to positive (Pos) controls; all other cells are labeled in black, corresponding to negative (Neg) controls. Channel 1 (Ch1) detected droplets Pos for the unmethylated target (FAM signal, blue color), and channel 2 (Ch2) detected droplets Pos for the methylated target (HEX signal, green color). The threshold used to discriminate between Pos and Neg droplets is shown as a purple line. (B) Number of droplets Pos for the unmethylated target corresponding to cell-free DNA (cfDNA) from endothelial cell origin. (C) Number of droplets Pos for the methylated target corresponding to cfDNA or DNA from nonendothelial cell origin. The ddPCR was performed using genDNA from control cells and cell lines (NTC, genDNA, PBMC, Neutro, HUVECs, HMEC1, and Octaplas).

Based on published data and open-source databases, we developed a methylation-specific ddPCR assay to quantify cfDNA originating from endothelial cells, targeting the promoter region of the *NOS3* gene. The *NOS3* gene encodes nitric oxide synthase 3 (also known as “eNOS” or constitutive NOS), which is specifically expressed in endothelial cells [22]. Unmethylated *NOS3* sequences serve as a marker for cfDNA originating from endothelial cells. In contrast, methylated *NOS3* sequences are found in all other cell types. In brief, we identified suitable regions with differentially methylated CpG sites in the *NOS3* promoter (Supplementary Figure S2) [[22], [23], [24], [25]]. The unmethylated PCR product (cfDNA from endothelial cell origin) was detected in channel 1 (TaqMan probe labeled with FAM) and the methylated PCR product (cfDNA from nonendothelial cells) in channel 2 (TaqMan probe labeled with HEX) using the QX200 Droplet Reader. After applying rigorous quality control to experiments using DNA from endothelial cell cultures (HUVECs and HMEC-1) and nonendothelial cells, we identified a methylation-specific *NOS3* ddPCR assay with an 84-bp amplicon that reliably distinguished cfDNA from endothelial cells from cfDNA from other cell types (Figure 1). A comprehensive description of the assay development process is provided in the Supplementary Methods.

### 3.2 Circulating total cfDNA levels in COVID-19 patients and healthy controls

**Figure 2:**
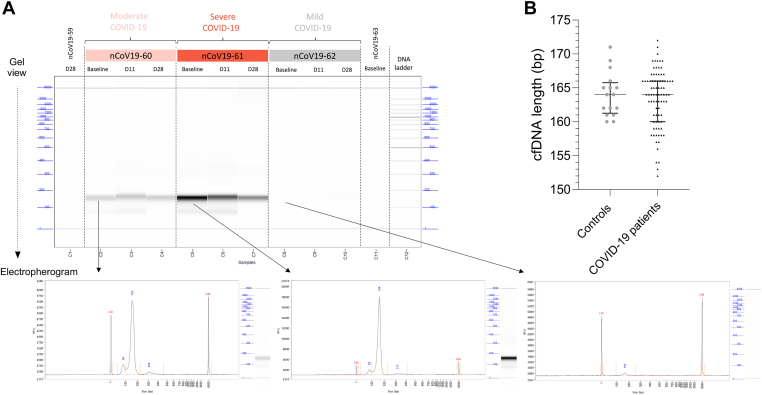
Fragment length of cell-free DNA (cfDNA). (A) Analysis of cfDNA from patients with different COVID-19 disease severity using a fragment analyzer, digital gel view ProSize data (Agilent) analysis software with samples from 5 different COVID-19 patients over time (top), and exemplary electropherograms of cfDNA from 3 COVID-19 patients with different disease severity, at baseline (bottom). (B) cfDNA length of interest from controls and all COVID-19 patients. Data are presented as median (IQR). Statistical significance is set at *P* < .05. Patients named nCOV19-60 (moderate), nCOV19-61 (severe), nCOV19-62 (mild) and nCOV19-63 (mild).

**Figure 3:**
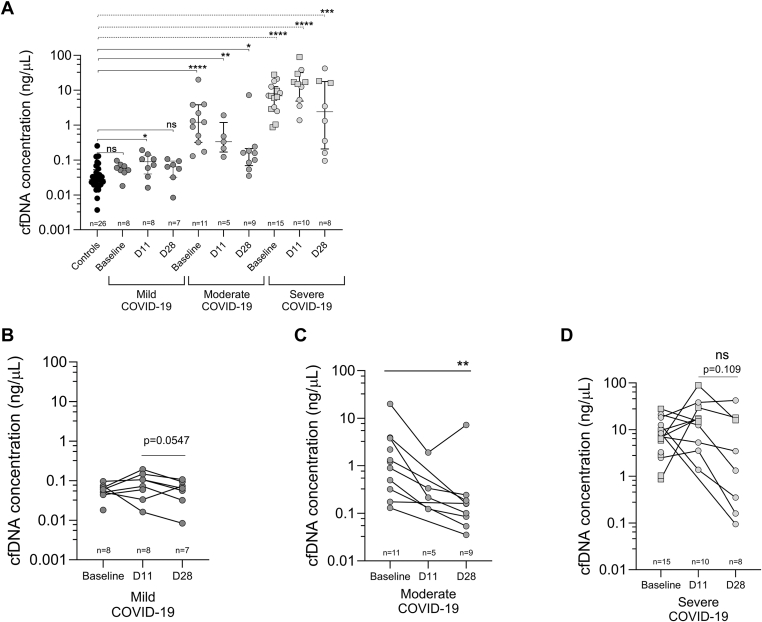
Plasma cell-free DNA (cfDNA) concentrations in COVID-19 patients and healthy controls (controls). (A) cfDNA concentration of 35 patients with COVID-19, split into different disease severity groups: mild (*n* = 9), moderate (*n* = 11), severe (*n* = 15), and controls (*n* = 26), measured using a fragment analyzer. For one patient with mild COVID-19, a baseline (day of enrollment) sample is missing, but samples collected 11 ± 3 days after enrollment (D11) and 28 ± 7 days after enrollment (D28) are available. (B) cfDNA concentration in samples of patients with mild COVID-19, (C) moderate COVID-19, and (D) severe COVID-19. Plasma samples from controls and patients infected with SARS-CoV-2 with varying disease severity (mild, moderate, and severe) were analyzed at baseline, D11, and D28. Squares represent patients who died during the study period. Data are presented as median (IQR). Statistical significance is set at *P* < .05. ns, not significant.

**Figure 4:**
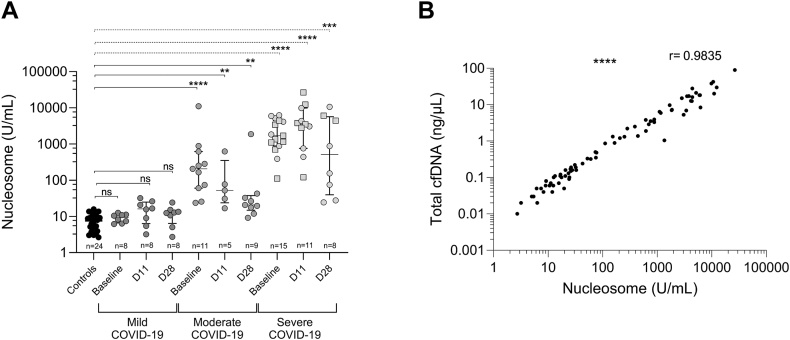
Nucleosome concentrations in COVID-19 patients and healthy controls. (A) Nucleosome concentration of 35 patients with COVID-19, split into different disease severity groups: mild (*n* = 9), moderate (*n* = 11), severe (*n* = 15), and healthy donors (control; *n* = 24). For one patient with mild COVID-19, a baseline (day of enrollment) sample is missing, but samples collected 11 ± 3 days after enrollment (D11) and 28 ± 7 days after enrollment (D28) are available. (B) Correlation curve between the total amount of cell-free DNA (cfDNA) quantified with a fragment analyzer and nucleosome levels in 35 patients with COVID-19; all time points are included (*n* = 81 pairs). Plasma from controls and patients infected with SARS-CoV-2 with varying disease severity (mild, moderate, and severe) was analyzed at baseline, D11, and D28. Squares represent patients who died during the study period. Data are presented as median (IQR). Statistical significance is set at *P* < .05. ns, not significant.

cfDNA was extracted from the plasma of healthy controls (*N* = 26) and COVID-19 patients (*N* = 35; Table). For a patient with mild COVID-19, only samples at D11 and D28 were available. In the electropherograms used for quality control after extraction, the cfDNA peaks of interest were observed within the 100 to 250 bp size range (Figure 2). The length of cfDNA fragments isolated from healthy controls and COVID-19 patients across all time points was similar (164 ± 6 bp vs 164 ± 4 bp; *P* > .05; Figure 2). Circulating total cfDNA levels were higher in patients with moderate and severe COVID-19 at baseline (moderate COVID-19: 1.2 ng/μL [0.3-3.8], *P* < .0001; severe COVID-19: 6.9 ng/μL [2.9-13], *P* < .0001), D11 (moderate COVID-19: 0.3 ng/μL [0.2-1.2], *P* = .004; severe COVID-19: 16 ng/μL [4.9-32], *P* < .0001), and D28 (moderate COVID-19: 0.2 ng/μL [0.07-0.2], *P* = .02; severe COVID-19: 2.4 ng/μL [0.2-17], *P* = .003) than in healthy controls (0.02 ng/μL [0.02-0.08]; Figure 3A). In contrast, patients with mild COVID-19 had only slight increases in total cfDNA levels (baseline: 0.06 ng/μL [0.05-0.07], *P* = .16; D11: 0.09 ng/μL [0.04-0.14], *P* = .01; D28: 0.06 ng/μL [0.03-0.09], *P* = .16) compared with healthy controls. COVID-19 patients with moderate disease had higher total cfDNA levels across all 3 time points compared with those with mild disease. Similarly, COVID-19 patients with severe disease had higher cfDNA concentrations than those with moderate disease (Figure 3A). We validated these findings by applying an alternative method for quantifying cfDNA release into circulation: measuring plasma nucleosome concentrations (Figure 4). Nucleosomes are the basic structural units of chromatin and can serve as an alternative marker of cfDNA release into plasma [[8], [9], [10]].

**Table:** Demographic characteristics of COVID-19 patients.

Characteristics	Mild disease (*n* = 9)		Moderate disease (*n* = 11)		Severe disease (*n* = 15)	
	*n*	%	*n*	%	*n*	%
**Sex**						
Female	5/9	56	1/11	9	1/15	7
Male	4/9	44	10/11	91	14/15	93
**Age group, y**						
18-49	9/9	100	3/11	27	1/15	7
≥50	0/9	0	8/11	73	14/15	93
**Age (y), median (range)**	29 (24-40)		61 (40-84)		69 (41-84)	
**Symptoms**						
Fever	3/9	33	8/11	73	11/12	92
Rhinorrhea	7/9	78	1/11	9	1/6	17
Sore throat	4/9	44	2/11	18	0/6	0
Cough	4/9	44	10/11	91	10/11	91
Dyspnea	1/9	11	5/11	45	10/11	91
Myalgia	5/9	56	3/11	27	2/7	29
Nausea	0/9	0	1/11	9	1/6	17
Diarrhea	1/9	11	4/11	36	4/6	27
Anosmia	-	-	1/1	100	-	-
**Duration of symptoms, d**						
**Baseline,** median (IQR)	4 (3-6)		8 (4-11)		7 (5-11)	
**D11,** median (IQR)	17 (15-17)		21 (16-25)		17 (14-22)	
**D28,** median (IQR)	32 (32-34)		37 (33-42)		29 (28-39)	
**Comorbidities**						
Diabetes	0/9	0	0/11	0	6/15	40
Cardiovascular	0/9	0	4/11	36	8/15	53
Hypertension	0/9	0	2/11	18	9/15	60
Pulmonary	1/9	11	2/11	18	1/15	7
Immune	0/9	0	1/11	9	2/15	13
Malignancy	0/9	0	1/11	9	5/15	33
Kidney	0/9	0	0/11	0	4/15	27
Other	0/9	0	4/11	40	3/15	20
At least 2 comorbidities	0/9	0	3/11	27	13/15	87
**Other information**						
Hospitalized	0/9	0	11/11	100	15/15	100
ICU at enrollment	0/9	0	1/11	9	13/15	87
ICU at any time	0/9	0	1/11	9	15/15	100
Death	0/9	0	0/11	0	8/15	53
**Treatment**						
No antiviral or anti-inflammatory therapy	9/9	100	6/11	55	0/15	0
Antiviral therapy	0/9	0	0/11	0	0/15	0
Corticosteroid therapy	0/9	0	5/11	45	15/15	100

Baseline, enrollment day; D11, 11 ± 3 days after enrollment; D28, 28 ± 7 days after enrollment; ICU, intensive care unit.

When analyzing intraindividual total cfDNA concentrations over the course of COVID-19 disease, we found that most patients with moderate COVID-19 showed steadily decreasing cfDNA levels from the time of diagnosis to D28 (Figure 3C), whereas patients with severe disease had higher baseline levels and may have had increasing cfDNA concentrations until D11 (Figure 3D). Patients with mild disease had low cfDNA concentrations at baseline that did not decrease substantially over the course of the disease (Figure 3B).

### 3.3 Circulating endothelial cell-specific cfDNA levels in COVID-19 patients and healthy controls

Next, we aimed to quantify the endothelial cell-specific cfDNA in healthy controls and COVID-19 patients. We excluded samples that showed no positive droplets in the ddPCR assay. Additionally, samples with only 1 or 2 positive droplets for both endothelial and nonendothelial targets were excluded, as these droplets were highly likely to be false positives (probability of being true positives < .004). We analyzed the data using 2 approaches: first, using raw values and reporting the number of ddPCR-positive droplets per sample; and second, converting the droplet counts to cfDNA concentrations using QuantaSoft software.

**Figure 5:**
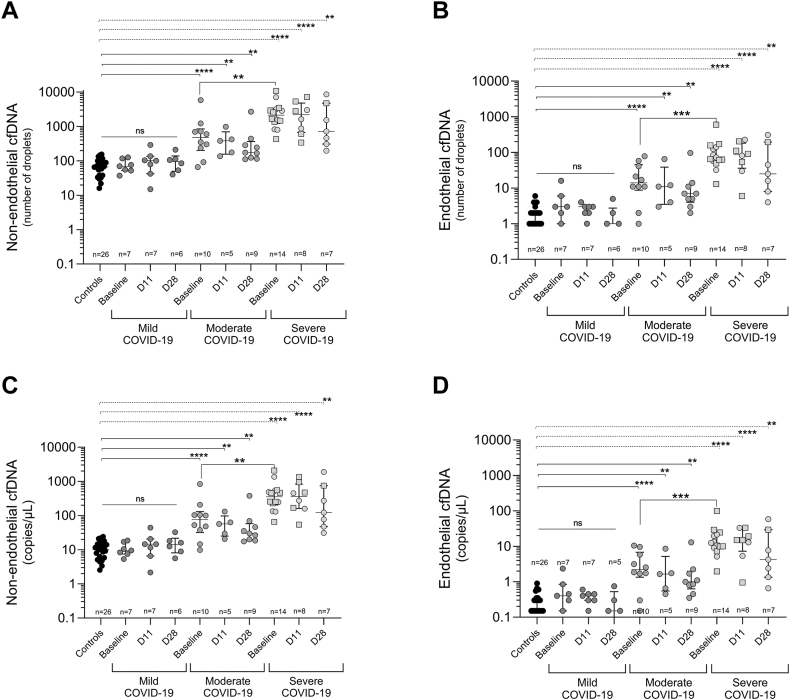
Assessment of endothelial and nonendothelial cell-free DNA (cfDNA) according to COVID-19 severity. (A) Number of positive droplets of the methylated target corresponding to cfDNA from all nonendothelial cells. (B) Number of positive droplets of the unmethylated target, corresponding to cfDNA from endothelial cells. (C) Concentration of nonendothelial cfDNA measured with a fragment analyzer. (D) Concentration of endothelial cfDNA in copies/μL, as calculated by the QuantaSoft software. Plasma from healthy donors (controls, *n* = 26) and from patients infected with SARS-CoV-2 with varying disease severity (mild [*n* = 7], moderate [*n* = 10], and severe [*n* = 14]) was analyzed at different time points: baseline, day of enrollment; D11, 11 ± 3 days after enrollment; and D28, 28 ± 7 days after enrollment. Eight samples (mild COVID-19: 1 at baseline, 1 on day 11, and 1 on day 28; moderate COVID-19: 1 at baseline; severe COVID-19: 1 at baseline, 2 on day 11, and 1 on day 28) were excluded from analysis because DNA amplification was unsuccessful. Squares represent patients who died during the study period. Data are presented as median (IQR). Statistical significance is set at *P* < .05. ns, not significant.

In patients with moderate and severe COVID-19, endothelial-specific cfDNA levels, as measured by the number of ddPCR-positive droplets, were elevated compared with healthy controls (*P* < .0001 for both). Specifically, in the moderate COVID-19 group, the levels were as follows: baseline: 14 [8.7-45]; D11: 11 [3.5-38]; and D28: 7 [4.0-13] positive droplets. For the severe COVID-19 group, the levels were as follows: baseline: 68 [55-146]; D11: 86 [36-182]; and D28: 25 [8-195] positive droplets. In healthy controls, the level was 1 [1.0-2.0] positive droplets (Figure 5). Patients with mild COVID-19 (baseline: 3 [1.0-6.0]; D11: 3 [2.0-3.0]; and D28: 1 [0.0-2.7] positive droplets) did not have higher circulating endothelial cell-specific cfDNA concentrations than healthy controls (Figure 5). Endothelial cfDNA showed a strong and significant correlation with total cfDNA (r = .8980; *P* < .001) and nucleosomes (r = .9034; *P* < .001).

Conversion of the droplet count to cfDNA concentration using QuantaSoft software did not change the results (Figure 5).

### 3.4 The proportion of circulating endothelial cell-specific cfDNA to cfDNA from other cell sources in relation to COVID-19 disease severity

Next, we aimed to investigate whether the proportion of endothelial cell-specific cfDNA relative to nonendothelial cell-derived cfDNA varies with the severity of COVID-19. A low number of positive droplets overall (endothelial and nonendothelial) might be an indicator of low sample quality and will lead to both higher false-positive rates and unreliable estimates of proportions, ultimately reducing discriminatory power. Therefore, we performed multiple analyses, excluding samples with very low positive droplet counts (sum of ddPCR-positive droplets for endothelial and nonendothelial cell-derived cfDNA) from the dataset in increments of 10 from 10 to 60, and selected the threshold at the point where the area under the curve (AUC) in the receiver operating characteristic (ROC) analysis began to plateau, resulting in a cutoff of 50 droplets.

**Figure 6:**
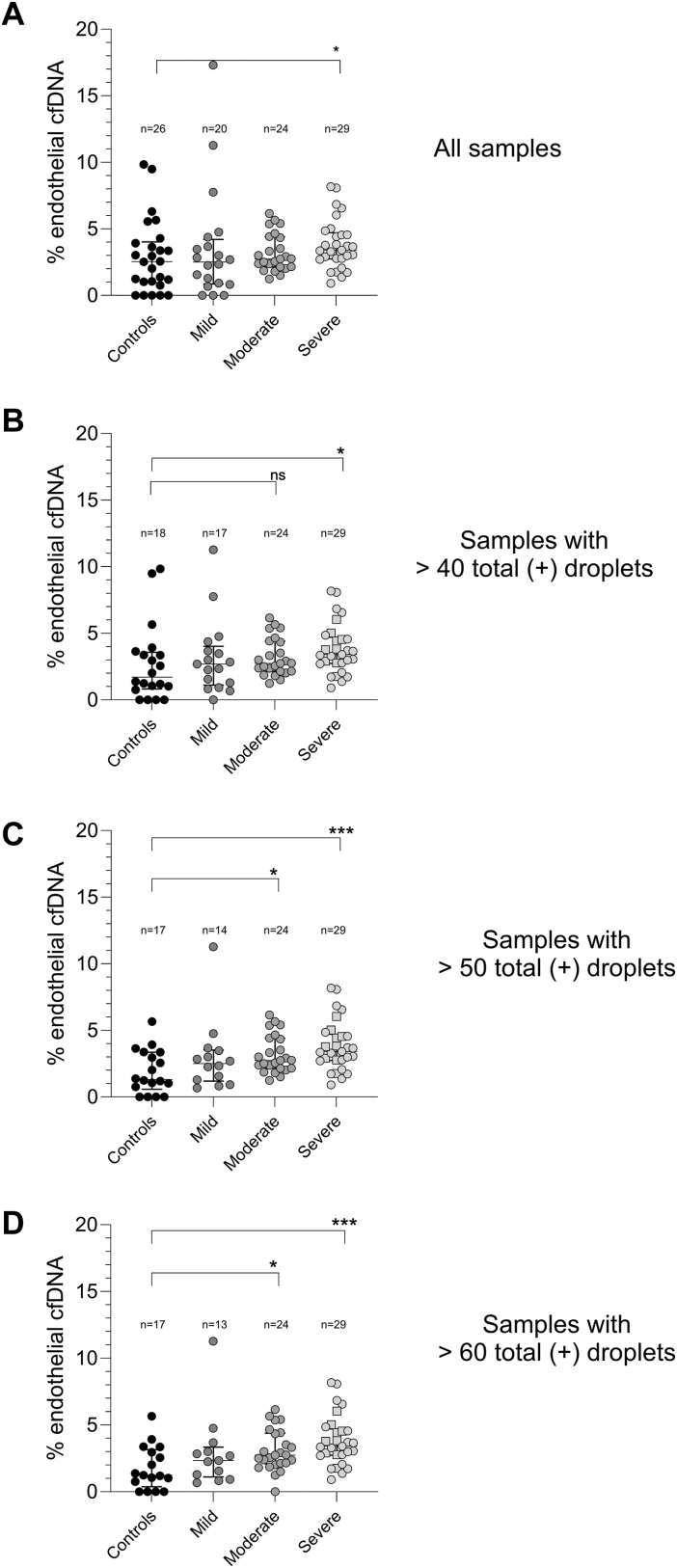
Quantification of the percentage of endothelial cell-free DNA (cfDNA) from the total amount of cfDNA in COVID-19 patients. (A) Percentage of droplets positive for endothelial cfDNA (unmethylated target) in all samples included. (B) Percentage of endothelial cfDNA when discarding all samples with <40 positive droplets in total. (C) Percentage of endothelial cfDNA when discarding all samples with <50 positive droplets in total. (D) Percentage of endothelial cfDNA when discarding all samples with <60 positive droplets in total. Plasma was collected from healthy donors (controls) and from 35 patients infected with SARS-CoV-2, with varying disease severity (mild, moderate, or severe), at all time points for each included individual. Squares depict patients who died during the study period. Data are presented as median (IQR). Statistical significance is set at *P* < .05. ns, not significant.

When all samples were included, the proportion of ddPCR-positive droplets for endothelial cell-derived cfDNA was elevated exclusively in patients with severe COVID-19 compared with healthy controls (Figure 6A). By stepwise excluding samples with low numbers of ddPCR-positive droplets, the proportion of endothelial cell-derived cfDNA increased in patients with moderate and severe disease (Figure 6B–D). As the threshold was raised, statistical significance improved, as indicated by a gradual decrease in the *P* value, though this came at the cost of a reduced sample size. Consequently, we considered a threshold of ≥50 ddPCR-positive droplets optimal, as it balances the need to avoid false positives with the inclusion of the maximum number of samples. ROC curve analysis confirmed these results (Supplementary Figure S4). Discrimination of disease severity levels, as measured by AUC, improved with a threshold of ≥50 total ddPCR-positive droplets compared with 40 droplets, but remained similar when set at 60 droplets.

Next, we analyzed the performance of various measures in discriminating among COVID-19 disease severity levels. Instead of using the proportion of ddPCR-positive droplets for endothelial cell-derived cfDNA, we looked at the absolute number of ddPCR-positive droplets for both endothelial cell-specific cfDNA and cfDNA from other sources, as well as the total number of positive droplets (sum of endothelial and nonendothelial; Supplementary Table S3). These alternative measures showed a higher AUC compared with the proportion of endothelial cell-derived cfDNA (Supplementary Tables S4 and S5).

Next, we evaluated whether cfDNA from endothelial cells increases disproportionately with disease severity, suggesting that endothelial cell damage is a primary contributor to disease progression. Indeed, the number of droplets positive for cfDNA of endothelial cell origin increased by a factor of 4.3 (rate ratio [RR], 4.3 [95% CI, 3.5-5.2]) per 1-step increase in disease severity, as rated by the COVID-19 WHO Ordinal Scale for Clinical Improvement. The number of droplets positive for cfDNA from nonendothelial cell origin only rose by a factor of 3.5 (RR, 3.5 [95% CI, 3.0-4.1]). The difference in RR was significant (*P* = .01).

## 4 Discussion

We developed a methylation-specific ddPCR assay to detect endothelial cfDNA targeting a region of the *NOS3* promoter, which is unmethylated in vascular endothelial cells but methylated in all other cell types. This assay was confirmed to be highly specific for endothelial cfDNA. Using this method, we demonstrated that endothelial cell-specific cfDNA levels increase with COVID-19 severity. As COVID-19 disease severity increased, the circulating total cfDNA concentration in plasma rose. Notably, as the severity of COVID-19 increased, the total concentration of circulating cfDNA in plasma also rose. Moreover, the proportion of cfDNA originating from endothelial cells increased, highlighting the significant role of endothelial cell damage in disease progression.

During assay establishment and validation, we demonstrated that our methylation-specific ddPCR assay is specific for detecting endothelial DNA. In samples containing DNA isolated from peripheral blood mononuclear cells, neutrophils, or pooled plasma, only DNA from nonendothelial sources (methylated DNA) was detected. In contrast, in samples containing DNA derived from HUVEC cultures or human microvascular endothelial cells-1 (an immortalized endothelial cell line), nearly all droplets detected by the ddPCR assay contained DNA of endothelial cell origin (unmethylated DNA), indicating strong specificity for endothelial-derived DNA. Using ddPCR offers several advantages over classical real-time quantitative PCR. ddPCR enables absolute DNA quantification with high precision, even when minimal amounts of template are available. It also exhibits high sensitivity and robust tolerance to PCR inhibitors. Moreover, ddPCR eliminates the need for calibration or standard curves, thereby simplifying the quantification process [26].

Recent findings from our group and data from the current study demonstrate that nuclear cfDNA plasma concentrations, measured by ddPCR or as nucleosomes, significantly correlate with disease severity in COVID-19 patients [17,27]. Previously, we and others have shown that plasma mitochondrial cfDNA (mtcfDNA) concentrations are associated with COVID-19 disease severity [17,27]. Andargie et al. [27] analyzed cfDNA in patients with COVID-19 by quantifying nuclear DNA and mtcfDNA using ddPCR, followed by bisulfite sequencing and analysis of cfDNA sequence reads using a deconvolution algorithm and a library of tissue-specific DNA methylation signatures to identify the cellular sources of cfDNA. In line with our results, total cfDNA was significantly increased in the plasma of COVID-19 patients compared with healthy controls, and the same was true for mtcfDNA. Interestingly, they showed elevated cfDNA concentrations from the vascular endothelium in the plasma of COVID-19 nonsurvivors compared with survivors [27]. Similarly, we also found that the concentration of cfDNA derived from endothelial cells is increased in patients with severe COVID-19. Additionally, we demonstrated that the concentration of cfDNA derived from endothelial cells increases proportionally in patients with severe COVID-19, indicating that these patients experience more pronounced endothelial cell damage. Our findings are consistent with those of previous autopsy studies, which describe endothelialitis in fatal COVID-19 [2,28]. Our study highlights the potential of circulating cfDNA as a valuable tool for liquid biopsies, a specialized technique used to analyze nonsolid biological tissues. It is important to underscore that although circulating endothelial cfDNA represents DNA originating from endothelial cells, this does not inherently imply that endothelial injury is the predominant mechanism driving COVID-19 pathogenesis.

A unique feature of our study is that we followed patients longitudinally, enabling us to assess the temporal dynamics of cfDNA concentration across patient groups with different disease severity. However, our study has important limitations that warrant discussion. While total cfDNA is elevated during severe infection, cell-type-specific cfDNA analysis provides information on tissue sources of injury. The observed increase in endothelial cfDNA supports the hypothesis that vascular damage contributes to COVID-19 pathogenesis. Future studies integrating endothelial cfDNA levels with clinical outcomes, such as multiorgan failure, need for ventilation, and long-term sequelae, will be essential to establish its prognostic relevance. The patients were included during the first and second waves of COVID-19, prior to widespread vaccine- or infection-induced adaptive immunity to SARS-CoV-2 in the population. While preexisting humoral and cell-mediated adaptive immunity to SARS-CoV-2 may not provide consistent protection against reinfection, it is likely to influence disease severity and the resulting endothelial cell damage. The influence of demographic and comorbidity-related factors on cfDNA levels cannot be excluded. The cohort size did not permit multivariate analysis adjusting for sex, age, or comorbidities, which may confound differences in cfDNA concentration. Our healthy control group consists solely of anonymous blood donors; consequently, we lack sex- and age-matched controls. We did not systematically screen for immunothrombotic pathologies in our cohort, nor did we conduct autopsies. Consequently, we are unable to provide data on clinically apparent vascular pathologies or autopsy findings, nor on their impact on the concentration of endothelial cell-specific cfDNA. Unfortunately, there was also no plasma left to measure plasma markers for endothelial cell activation, such as von Willebrand factor antigen, thrombomodulin, P-selectin, or endothelin-1. Future prospective studies integrating cfDNA quantification with plasma biomarkers of endothelial activation (eg, von Willebrand factor, P-selectin, and thrombomodulin) could further elucidate the link between cfDNA release and vascular injury [29,30].

In summary, we established a highly specific ddPCR assay for the detection and quantification of cfDNA derived from endothelial cells. Applying this method, we demonstrated that severe COVID-19 is associated with endothelial cell damage, as indicated by increasing endothelial cell-specific cfDNA concentrations in plasma. We demonstrated that host-derived cfDNA is a valuable biomarker for quantifying damage resulting from host-pathogen interactions in infectious diseases.
