# Supplementary material for: Quantifying endothelial damage by digital droplet polymerase chain reaction (PCR) of endothelial cell-free DNA in COVID-19 patients
Source: Res Pract Thromb Haemost. 2026 Jan 13;10(1):103320. doi: 10.1016/j.rpth.2025.103320 (PMC12925348; doi:10.1016/j.rpth.2025.103320)
Supplement: Supplementary Material [file mmc1.docx]

**SUPPLEMENTARY INFORMATION**

**Endothelial Cell-Free DNA Release Depending on COVID-19 Severity in Droplet Digital PCR Analysis with *NOS3* Gene Methylation Status**

Tiphaine Ruggeri, Gertrud Wiedemann, Noëlia Schärz, Barbara Hügli, Andreas Limacher, Cédric Hirzel, Naomi Porret and Sacha Zeerleder

**Supplementary Methods**

**Cell cultures**

Human dermal microvascular endothelial cells (HMEC-1) and human umbilical vein endothelial cells (HUVEC) were obtained from Lonza (Breda, The Netherlands) and cultured in endothelial cell culture medium EGM^TM^ -2 MV Microvascular Endothelial Cell Growth Medium-2 BulletKit^TM^ (CC-3202, Lonza). Cells were subcultured until passage six before extracting DNA.

**Neutrophil and PBMC isolation**

Neutrophils were isolated from 9 mL peripheral heparinized blood collected from healthy donors. The density gradient was prepared using Histopaque®-1119 (Sigma-Aldrich, USA) and Ficoll-Paque® PLUS (GE Healthcare, USA). The sample was diluted 1:2 in PBS (Fresenius Kabi, Germany) and layered on top of the density gradient, then centrifuged for 25 min at 1500 rpm. The neutrophil layer was collected and diluted in Iscove's Modified Dulbecco's Medium (Lonza) supplemented with 5% fetal calf serum, then centrifuged twice at 1500 rpm for 5 min in order to obtain the neutrophil pellet.

Peripheral blood mononuclear cells (PBMCs) were isolated from 9 mL EDTA blood, also using the Ficoll method (Ficoll Lymphoprep Density 1.077g/mL, Nycomed, Switzerland).

**Workflow for measurement of total and endothelial cell specific cfDNA from plasma sampels**

The workflow from plasma sampling to performance of the ddPCR is illustrated in Supplemental Figure 1.

**
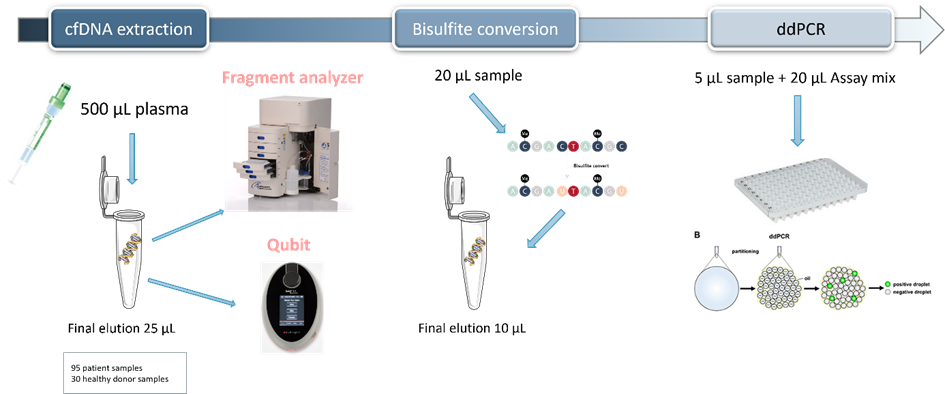
**

**Supplementary Figure S1. Design of the experiment with all steps from plasma sampling to performance of the ddPCR.**

cfDNA extraction (left), bisulfite conversion of cfDNA and controls (middle), ddPCR (right). DNA concentration and fragment analysis.

**DNA concentration and fragment analysis**

Total DNA concentration was measured using the Qubit® dsDNA HS (High Sensitivity) Assay Kit for the Qubit® 2.0 fluorometer (Invitrogen, USA). In addition, DNA samples were analyzed in the fragment analyzer (Advanced Analytical Technologies, USA) with the HS NGS Fragment 500 4C Kit (range of DNA size: 1-6000 bp) (Agilent Technologies, USA).

**Bisulfite conversion**

Bisulfite conversion of the samples was performed using the EpiTect® Plus DNA Bisulfite Kit (Qiagen, Germany) according to the manufacturer's protocol. Bisulfite reactions were prepared using 20 µL cfDNA in a total volume of 140 µL, as described in the manufacturer's protocol for low-concentration samples (1 – 500 ng DNA). For the control genDNA from cells, bisulfite reactions contained 200 ng DNA in a total volume of 140 µL reaction mix. Bisulfite conversion was performed in a Veriti 60 Well Thermal Cycler (Applied Biosystems, USA). After bisulfite conversion, patient and healthy donor cfDNA samples (including Octaplas) were eluted in 10 µL elution buffer, then split in two wells for ddPCR with 5 µL used per well. Control samples, i.e. genDNA from cells, were eluted in 15 µL elution buffer and the final volume was adjusted to 40 µL with distilled water. All samples were stored at -20°C until use.

**Design and optimization of methylation-specific ddPCR primers and probes for cfDNA originating from endothelial cells**

Based on published data and open-source databases, we designed a ddPCR assay to detect and quantify cfDNA originating from endothelial cells, targeting the promoter region of the *NOS3* gene. The *NOS3* gene, encoding for the nitric oxide synthase 3, also known as *eNOS* or constitutive *NOS* (*cNOS*), is located on chromosome 7, at position 150,991,017 to 151,014,588 in the genome assembly GRCh38/hg38 or from 15,690,841 to 150,701,023 in Genome Browser GRCh37/hg19. We aimed to detect unmethylated *NOS3* sequences as a marker for cfDNA originating from endothelial cells to facilitate discrimination from methylated *NOS3* sequences originating from all other cell types. For the *NOS3* promoter, the information available on the methylation status in the human genome browser for PBMC and cell lines (Supplemental Fig. 2a, b) corresponded exactly to the CpG sites identified by Chan and his collaborators [1].


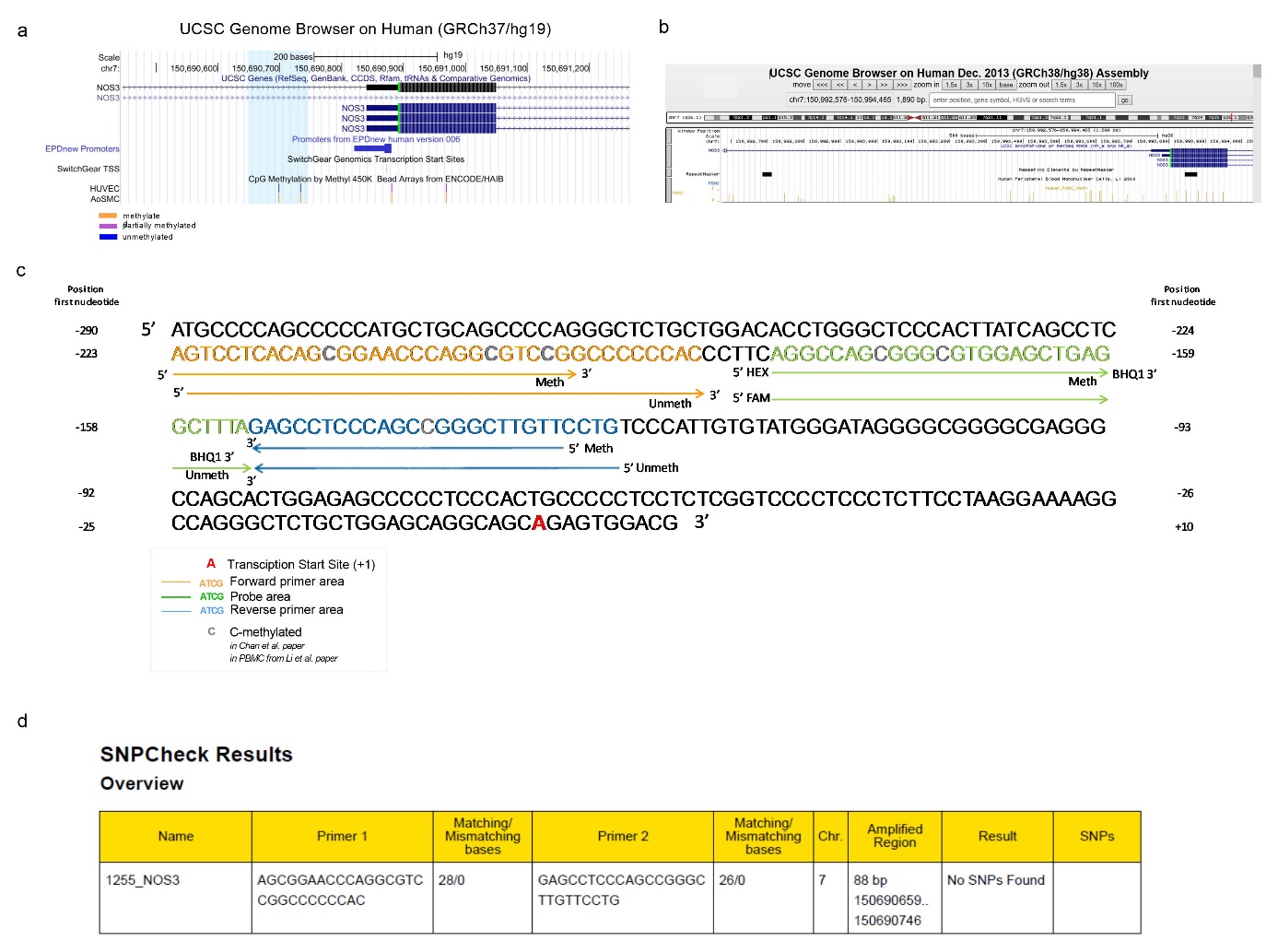


**Supplementary Figure S2.** Design *NOS3* (short version) primers and probes for methylation-specific ddPCR assay

**a**, Localization of NOS3 gene, its promoter, TSS and CpG sites methylated in cells lines (HUVEC and AoSMC) from Genomic browser in human GRCh37/hg19. **b**, Localization of CpG sites methylated in the NOS3 promoter in PBMC from Genomic browser in human GRCh38/hg38. **c**, Exact binding sites of primers and probes for NOS3 specific assay (short version). **d**, SNPcheck results for the primers designed. The A shown in red bold corresponds to the transcription start site (+1), orange sequences depict the forward primers, green sequences the probes, blue sequences reverse primers, C shown in grey bold corresponds to the methylated bases; Sequence sense from 5’ to 3’; sequence non-sense from 3’ to 5’.

HUVEC: human umbilical vein endothelial cells, AoSMC: human aortic smooth muscle cells, Meth: methylated, PBMC: peripheral blood mononuclear cells, SNP: single nucleotide polymorphism, Unmeth: unmethylated.

The first step was to design primers and probes including CpG (C-methylated) sites using several critical parameters to achieve the best possible assay performance and specificity to the sequence targeted (Supplemental Table 1). Based on published data, we identified suitable regions with differentially methylated CpG sites in the *NOS3* promotor [1-4]. We designed a first ddPCR assay targeting the selected sequence in *NOS3* resulting in an amplicon length of 145 bp (Supplemental Table 2).

| **Parameters** | **Rules** | **Websites** |
| --- | --- | --- |
| **Design primers** | In all the target sequence for having a first idea | https://www.urogene.org/methprimer/ |
| **Amplicon Length** | <150bp | - |
| **CpG site (C-methylated)** | In each primer and probe | - |
| **Primers length** | 20bp-30bp | - |
| **SNPs** | None in primers neither in probes | https://genetools.org/SNPCheck/snpcheck.htm |
| **Tm for primers** | 50°C-55°C | - |
| **Tm for probes** | >3°C to 10°C compared to Tm primers | - |
| **Probes sequences** | No G at 5’ end | - |
| **Structure** | No complementarity  No Hairpin  No self-annealing | biotools.nubic.northwestern.edu/OligoCalc.html |

**Supplementary Table S1.** Summary of rules followed to design primers and probes for the methylation specific ddPCR with website links used.

CpG: Cytosine phosphate guanine, SNPs: Single nucleotide polymorphisms, Temperature melting: Tm

| **Gene** | **Sequence** |  | **Length** | **Final concentration** |
| --- | --- | --- | --- | --- |
| *NOS3* _meth_ | Forward primer | 5'- AGCGGAATTTAGGCGTTCG -3' | 19 bp | 700 nM |
| (short version) | Reverse primer | 5'- AACAAACCCGACTAAAAAACTC -3' | 22 bp | 700 nM |
|  | Probe | 5'- HEX-AGGTTAGCGGGCGTGGAGTTGAG-BHQ1 -3' | 23 bp | 200 nM |
| *NOS3* _unmeth_ | Forward primer | 5’- GTGGAATTTAGGTGTTTGGTTTTTTA -3’ | 26 bp | 1 400 nM |
| (short version) | Reverse primer | 5’- CAAAAACAAACCCAACTAAAAAACTC -3’ | 26 bp | 1 400 nM |
|  | Probe | 5’- FAM-AGGTTAGTGGGTGTGGAGTTGAGGTTTTA–BHQ1 -3’ | 29 bp | 400 nM |
| *NOS3* _meth_ | Forward primer | 5'- TTTTTATAGCGGAATTTAGGCGTT -3' | 24 bp | 700 nM |
| (long version) | Reverse primer | 5'- CCAATACTAACCCTCGCC -3' | 18 bp | 700 nM |
|  | Probe | 5'- HEX-AGGTTAGCGGGCGTGGAGTTGAG-BHQ1 -3' | 23 bp | 200 nM |
| *NOS3* _unmeth_ | Forward primer | 5’- GTTTTTATAGTGGAATTTAGGTGTTT -3’ | 26 bp |  |
| (long version) | Reverse primer | 5’- ACTCTCCAATACTAACCCTCA -3’ | 21 bp |  |
|  | Probe | 5’- FAM-AGGTTAGTGGGTGTGGAGTTGAGGTTTTA–BHQ1 -3’ | 29 bp |  |
|  |  |  |  |  |

**Supplementary Table S2.** Primers and probes for amplification of *NOS3* short and long versions for detection of methylated (meth) and unmethylated (unmeth) target sequences after bisulfite conversion.

After initial testing, we decided to design a second, optimized version of the methylation-specific ddPCR assay targeting *NOS3*. We chose primer locations resulting in the generation of a shorter amplicon length than the first (long) version as many cfDNA molecules in the samples will not serve as templates in the ddPCR, because the fragments are too short to span the entire length of the assay. To enhance the *NOS3* ddPCR assay performance for detection of small cfDNA fragments, we adjusted the design by positioning the primer closer to the probe, targeting an amplicon size of 84 bp (short version) (Supplemental Figure 2c, d). We ensured that none of the primer and probe sequences overlap with single nucleotide polymorphisms (SNPs).

To compare the performance of the two assay design versions (long version, 145 bp amplicon size vs short version, 84 bp amplicon size), genDNA from human umbilical vein endothelial cells (HUVECs; positive control) and from neutrophils (negative control) were used [1]. A temperature gradient experiment was conducted to determine the optimal annealing temperature, resulting in the best cluster separation of positive and negative droplets in both channels. We used the shorter version for the following experiments, as it resulted in a better separation between positive and negative droplets in both channels (Supplemental Figure 3).

To assess the specificity of the assay and to define the thresholds to be set for separation of positive and negative droplets in both channels, different positive and negative controls, including both cfDNA and genDNA, were tested. In channel 1, detecting the FAM- positive unmethylated target, the threshold was set at an amplitude of 12800, and for channel 2, detecting the HEX-positive methylated target, at 4000 (Figure 1a of the main manuscript). In channel 1, an increased number of positive droplets was detected (unmethylated target) in samples containing DNA extracted from HUVECs and human dermal microvascular endothelial cells (HMEC-1), the positive controls for endothelial cells. In channel 2, reflecting the methylated targets, only a low number of droplets were detected for DNA extracted from HUVECs and HMEC-1 (Figure 1b of the main manuscript). In contrast, only a very low number of droplets in the samples containing DNA extracted from peripheral blood mononuclear cells (PBMCs), commercial pool plasma (Octaplas), genomic DNA (genDNA) or a no template control (NTC) could be detected in channel 1 (unmethylated target; Figure 1b of the main manuscript). In channel 2, an increased number of droplets reflecting methylated amplicons, originating from non-endothelial cell types, could be detected in the samples containing DNA extracted from PBMCs, Octaplas as well as genDNA (Figure 1c of the main manuscript). These results demonstrate that the ddPCR assay is specific for the detection of genDNA and cfDNA originating from endothelial cells, by targeting a selected region of the *NOS3* promoter.

**Digital droplet PCR**

Digital droplet PCR (ddPCR) targeting methylated or unmethylated sequences in the promoter region of the *NOS3* gene was performed using the QX200 droplet digital PCR system (Bio-Rad, USA) including an AutoDG Automated Droplet Generator. Before ddPCR was performed, genDNA extracted from cells was digested with the restriction enzyme MseI (New England Biolabs, UK) for 15 min at room temperature, a step not necessary for already small fragment-sized cfDNA samples. Amplification reactions for ddPCR were prepared according to the manufacturer’s protocol, adding 5 µL bisulfite converted DNA to 20 µL assay mix. The input amount of template per ddPCR well was half of the cfDNA obtained from each sample after bisulfite conversion or 25 ng genomic DNA after restriction digest for cells, with all samples measured in duplicate. For automated droplet with the AutoDG Automated Droplet Generator 20 µL of the reaction mix was used for each well. Amplification took place in a C1000 Touch™ Thermal Cycler (Bio-Rad, USA) with the following thermal conditions: 95°C for 10 min, followed by 40 cycles of 94°C for 30 s, a combined step for annealing and amplification at 58°C for 1 min and a final deactivation step at 98°C for 10 min. All steps were performed with a ramp rate of 2°C/s. For readout the QX200 Droplet Reader with the two-color detection system set to FAM (channel 1) and HEX (channel 2) was used. Sequences and concentrations of primers and probes, as well as the ddPCR thermocycling conditions are summarized in Supplemental Tables 1 and 2.

**Data analysis of ddPCR**

Results were analyzed using the QuantaSoft software (Version 1.7.4.0917, Bio-Rad). Thresholds between positive and negative droplets were determined using the controls from Octaplas, cell lines and set at an amplitude 12800 for channel 1 (FAM, unmethylated PCR product) and 4000 for channel 2 (HEX, methylated PCR product) (Supplemental Figure 3) to separate positive droplets from the background. For all the samples, the QuantaSoft software provides us the number of positive droplets detected in channel 1 (unmethylated target) and channel 2 (methylated target), and the calculation of DNA concentration. For all samples, we calculated the percentage of droplets positive for the unmethylated target in channel 1 from all positive droplets (channel 1 and channel 2), which is the proportion of endothelial cfDNA or DNA, respectively:

*E% = E unmethylated / (E unmethylated + E methylated)*100.*


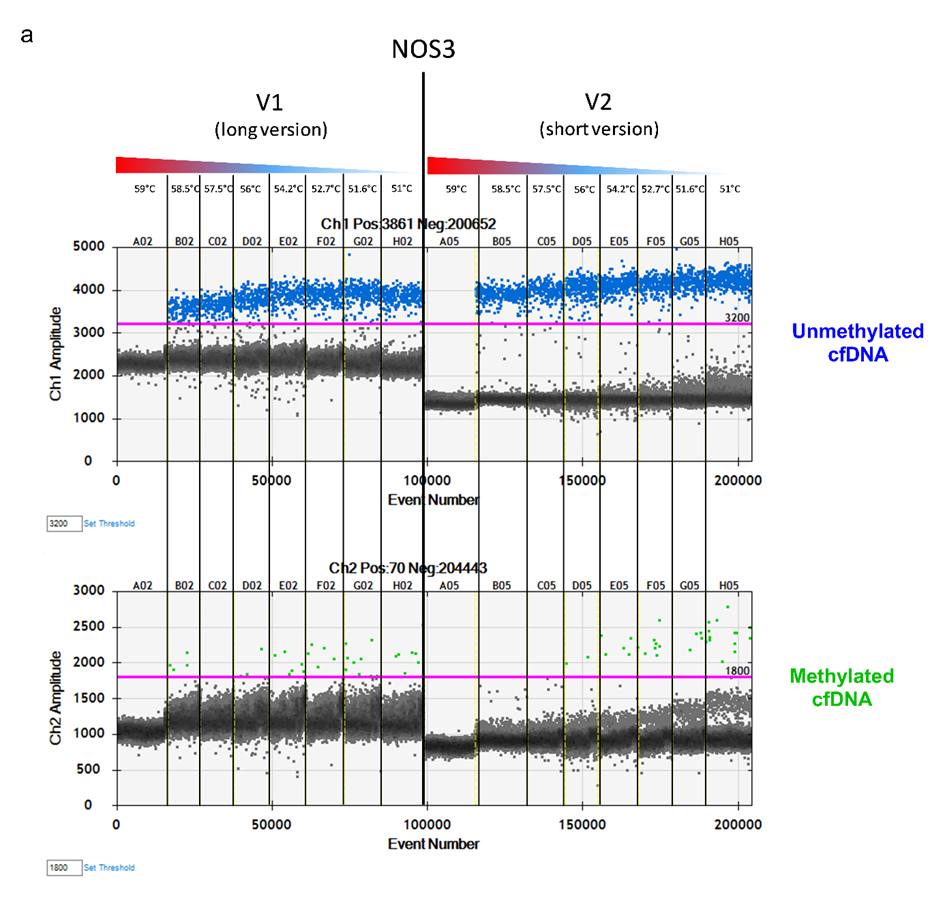


**Supplementary Figure S3.** ddPCR comparison of two the two different *NOS3* targeting assays designed using temperature gradient

**a**, 1D plot view from QuantaSoft software showing the droplet signal of HUVECs cells lines with two versions of the NOS3 targeting assays designed: V1 the longer version with 145bp and V2 the short version with 84bp amplicon size, respectively. The gradient is from the highest to the lowest temperature as following: 59°C, 58.5°C, 57.5°C, 56°C, 54.2°C, 52.7°C, 51.6°C and 51°C. In channel 1, droplets positive for the unmethylated cfDNA has been measured (FAM signal, blue color) and in the channel 2 droplets positive for the methylated cfDNA (HEX signal, green color). The threshold set to discriminate positive from negative droplets is shown as purple line. ddPCR: digital droplet polymerase chain reaction, HUVECs: human umbilical vein endothelial cells.

**Statistical Analysis**

For non-paired analysis (comparison of cfDNA concentrations among COVID-19 disease categories at same time points), we used Mann-Whitney test (comparison between two COVID-19 disease categories) or Kruskal-Wallis test followed by the Dunn’s multiple comparison test (comparison between >2 COVID-19 disease categories). For paired analysis (comparison of cfDNA concentrations at two different time points within COVID-19 disease categories) we applied the Wilcoxon signed rank test. Correlations were assessed by using Spearman’s rank correlation.

Samples with a total of two or fewer positive droplets were excluded (n = 12), leaving 98 samples of a total of 61 participants (35 patients and 26 controls). Data for the number of positive droplets and percentage of droplets positive for the unmethylated target across severity groups are presented as median with quartiles and mean with standard deviation. The different severity groups, as defined above, were compared by a non-parametric test of trend according to Cuzick and a Wald test from a regression model with cluster robust standard errors, respectively. The increase in number of positive droplets across severity groups was calculated from a multivariate negative binomial model with two independent variables and cluster robust standard errors to account for multiple samples per participant. The WHO ordinal severity scale was used as independent variable, the number of droplets positive for the unmethylated and methylated target as dependent variables.

To evaluate the discriminative power of the total number of positive droplets (ddPCR) as well as the percentage of droplets positive for the unmethylated target for patients with and without COVID-19 disease as well as patients with more versus less severe disease, an ROC curve analysis was performed. For the area under the ROC curve (AUC), a bootstrap 95% confidence interval and p-value was calculated to account for multiple samples per participant. Moreover, the odds ratio with 95% confidence interval and p-value was calculated per increase in one log10-unit of the number of positive droplets and in one percent unit of droplets positive for the unmethylated target from a logistic model. To account for clustering of data, cluster robust standard errors were used. Analyses were done in Stata version 17 (Stata Corp., College Station, TX, USA) and GraphPad Prism software version 9.3 (GraphPad Software, USA).

**Supplementary Results**

**Supplementary Figure S4. Proportion of positive droplets for cfDNA deriving from endothelial cells**


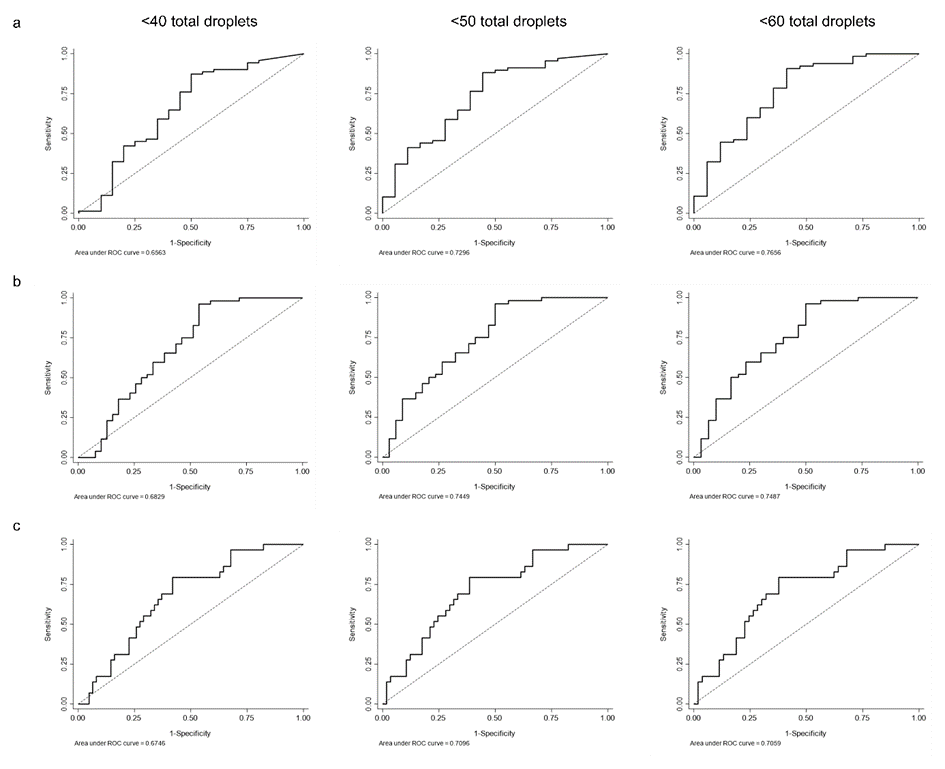


ROC curves with area under the ROC curve (AUC) for samples having less than 40 positive total droplets discarded (top), samples with less than 50 positive total droplets discarded (middle) and samples having less than 60 positive total droplets discarded (bottom) a, cases corresponding to all COVID-19 patients, versus controls. b, moderate or severe COVID-19 versus controls or mild COVID-19. c, severe COVID-19 versus controls, mild or moderate COVID-19. (+) positive

**Supplementary Table S3. Summary of the number of positive droplets in total, droplets positive for cfDNA deriving from endothelial cell**

|  | Control | Mild | Moderate | Severe |  |
| --- | --- | --- | --- | --- | --- |
| N | 26 | 20 | 23 | 29 |  |
|  | Median (quartiles) | Median (quartiles) | Median (quartiles) | Median (quartiles) | p-value |
| Number of total positive droplets | 68.5 (42.0;95.0) | 83.5 (53.5;119.5) | 313.0 (140.0;716.0) | 1726.0 (854.0;3306.0) | <0.001 |
| Number of positive droplets of unmethylated DNA | 1.0 (1.0;2.0) | 2.0 (1.0;3.0) | 11.0 (4.0;21.0) | 65.0 (32.0;141.0) | <0.001 |
| Number of positive droplets of methylated DNA | 66.5 (38.0;94.0) | 82.0 (52.5;116.0) | 302.0 (137.0;699.0) | 1631.0 (803.0;3181.0) | <0.001 |

Cell-free DNA samples were taken from control persons (healthy donors) and patients with COVID-19 at different severity: mild, moderate and severe. The unmethylated DNA corresponds to endothelial and the methylated DNA ton non-endothelial cell DNA. Data are presented as median (quartiles). Statistical significance p<0.05.

**Supplementary Table S4. Proportion of cfDNA of endothelial cell origin for different disease severity of COVID-19**

< 40 total positive droplets discarded

|  | Control | Mild | Moderate | Severe |  |
| --- | --- | --- | --- | --- | --- |
| N | 20 | 19 | 23 | 29 |  |
|  | Median (quartiles) | Median (quartiles) | Median (quartiles) | Median (quartiles) | p-value* |
| Percent unmeth positives [%] | 1.65 (0.90;3.39) | 2.30 (0.87;3.80) | 2.79 (2.14;4.50) | 3.69 (2.84;4.91) | 0.002 |
| Percent unmeth conc. [%] | 1.70 (0.89;3.51) | 2.34 (0.83;3.67) | 2.76 (2.15;4.43) | 3.41 (2.77;4.56) | 0.003 |
|  | Mean (SD) | Mean (SD) | Mean (SD) | Mean (SD) | p-value** |
| Percent unmeth positives [%] | 2.63 (2.81) | 2.84 (2.82) | 3.25 (1.45) | 3.95 (1.91) | 0.046 |
| Percent unmeth conc. [%] | 2.67 (2.85) | 2.83 (2.83) | 3.21 (1.43) | 3.82 (1.88) | 0.080 |

* p-value from Cuzick test; ** p-value from Wald test

< 50 total positive droplets discarded

|  | Control | Mild | Moderate | Severe |  |
| --- | --- | --- | --- | --- | --- |
| N | 18 | 16 | 23 | 29 |  |
|  | Median (quartiles) | Median (quartiles)) | Median (quartiles) | Median (quartiles) | p-value* |
| Percent unmeth positives [%] | 1.30 (0.74;3.28) | 2.30 (0.90;3.29) | 2.79 (2.14;4.50) | 3.69 (2.84;4.91) | <0.001 |
| Percent unmeth conc. [%] | 1.31 (0.76;3.35) | 2.30 (0.88;3.24) | 2.76 (2.15;4.43) | 3.41 (2.77;4.56) | <0.001 |
|  | Mean (SD) | Mean (SD) | Mean (SD) | Mean (SD) | p-value** |
| Percent unmeth positives [%] | 1.87 (1.61) | 2.62 (2.70) | 3.25 (1.45) | 3.95 (1.91) | <0.001 |
| Percent unmeth conc. [%] | 1.90 (1.64) | 2.60 (2.69) | 3.21 (1.43) | 3.82 (1.88) | <0.001 |

* p-value from Cuzick test; ** p-value from Wald test

< 60 total positive droplets discarded

|  | Control | Mild | Moderate | Severe |  |
| --- | --- | --- | --- | --- | --- |
| N | 17 | 13 | 23 | 29 |  |
|  | Median (quartiles) | Median (quartiles) | Median (quartiles) | Median (quartiles) | p-value* |
| Percent unmeth positives [%] | 1.27 (0.74;2.82) | 2.30 (1.25;2.88) | 2.79 (2.14;4.50) | 3.69 (2.84;4.91) | <0.001 |
| Percent unmeth conc. [%] | 1.23 (0.76;2.95) | 2.34 (1.29;3.01) | 2.76 (2.15;4.43) | 3.41 (2.77;4.56) | <0.001 |
|  | Mean (SD) | Mean (SD) | Mean (SD) | Mean (SD) | p-value** |
| Percent unmeth positives [%] | 1.77 (1.61) | 2.94 (2.79) | 3.25 (1.45) | 3.95 (1.91) | <0.001 |
| Percent unmeth conc. [%] | 1.79 (1.63) | 2.93 (2.78) | 3.21 (1.43) | 3.82 (1.88) | 0.001 |

* p-value from Cuzick test; ** p-value from Wald test

Tables with median ± quartiles and mean (SD) for controls and COVID-19 samples at mild, moderate and severe COVID-19, discarding samples having less than 40 positive total droplets (top), less than 50 positive total droplets (middle) or less than 60 positive total droplets (bottom). Unmethlyted DNA corresponds to the endothelial cell DNA and unmethylated DNA to non-endothelial cell DNA.

**Supplementary Table S5. Number of total positive droplets, droplets positive for cfDNA of endothelial origin and for cfDNA of non-endothelial cell origin.**

|  | OR (95%-CI) | p-value | AUC (95%-CI) | p-value |
| --- | --- | --- | --- | --- |
| Number of total positive droplets | | | | |
| Case vs control | 46.22 (6.19 to 344.95) | <0.001 | 0.87 (0.78 to 0.96) | <0.001 |
| Moderate/severe vs none/mild | 2328.73 (43.22 to 125459.29) | <0.001 | 0.96 (0.93 to 1.00) | <0.001 |
| Severe vs none/mild/moderate | 46.57 (5.29 to 410.23) | 0.001 | 0.95 (0.89 to 1.00) | <0.001 |
| Number of positive droplets for cfDNA of endothelial cell origin | | | | |
| Case vs control | 41.01 (7.86 to 213.94) | <0.001 | 0.87 (0.79 to 0.95) | <0.001 |
| Moderate/severe vs none/mild | 441.82 (24.32 to 8026.13) | <0.001 | 0.95 (0.90 to 1.00) | <0.001 |
| Severe vs none/mild/moderate | 36.94 (7.49 to 182.24) | <0.001 | 0.94 (0.88 to 1.00) | <0.001 |
| Number of positive droplets for cfDNA of non-endothelial cell origin | | | | |
| Case vs control | 41.95 (5.70 to 308.88) | <0.001 | 0.87 (0.78 to 0.96) | <0.001 |
| Moderate/severe vs none/mild | 2404.54 (44.10 to 131094.29) | <0.001 | 0.96 (0.93 to 1.00) | <0.001 |
| Severe vs none/mild/moderate | 47.07 (5.21 to 425.59) | 0.001 | 0.95 (0.89 to 1.00) | <0.001 |

Odd ratios (OR) and area under the ROC curve (AUC) analysis comparing case (all COVID-19 patients) versus control; moderate and severe diseases versus none and mild disease; and severe disease versus control (none), mild and moderate diseases. ORs are expressed per one log10-unit increase.

**References**

1 Chan Y, Fish JE, D'Abreo C, Lin S, Robb GB, Teichert AM, Karantzoulis-Fegaras F, Keightley A, Steer BM, Marsden PA. The cell-specific expression of endothelial nitric-oxide synthase: a role for DNA methylation. *J Biol Chem*. 2004; **279**: 35087–100. 10.1074/jbc.M405063200.

2 Li Y, Zhu J, Tian G, Li N, Li Q, Ye M, Zheng H, Yu J, Wu H, Sun J, Zhang H, Chen Q, Luo R, Chen M, He Y, Jin X, Zhang Q, Yu C, Zhou G, Sun J, Huang Y, Zheng H, Cao H, Zhou X, Guo S, Hu X, Li X, Kristiansen K, Bolund L, Xu J, Wang W, Yang H, Wang J, Li R, Beck S, Wang J, Zhang X. The DNA methylome of human peripheral blood mononuclear cells. *PLoS Biol*. 2010; **8**: e1000533. 10.1371/journal.pbio.1000533.

3 Shirodkar AV, St Bernard R, Gavryushova A, Kop A, Knight BJ, Yan MS, Man HS, Sud M, Hebbel RP, Oettgen P, Aird WC, Marsden PA. A mechanistic role for DNA methylation in endothelial cell (EC)-enriched gene expression: relationship with DNA replication timing. *Blood*. 2013; **121**: 3531–40. 10.1182/blood-2013-01-479170.

4 Chen C, Peng H, Huang X, Zhao M, Li Z, Yin N, Wang X, Yu F, Yin B, Yuan Y, Lu Q. Genome-wide profiling of DNA methylation and gene expression in esophageal squamous cell carcinoma. *Oncotarget*. 2016; **7**: 4507–21. 10.18632/oncotarget.6607.
